# Inbreeding and inbreeding depression of *Paeonia decomposita* (Paeoniaceae), a threatened endemic plant to China

**DOI:** 10.1186/s40529-019-0276-8

**Published:** 2019-12-05

**Authors:** Shi-Quan Wang

**Affiliations:** Ministry of Education Key Laboratory for Ecology of Tropical Islands, College of Life Sciences, Hainan Normal University, Haikou, 571158 China

**Keywords:** *Paeonia decomposita*, Reproductive biology, Conservation, Inbreeding, Inbreeding depression, Seed set

## Abstract

**Background:**

Small populations are predominantly vulnerable to inbreeding and inbreeding depression (ID). Owing to increased levels of inbreeding on individuals in small populations, ID could decrease the population growth rate, as well as its effective size, and exacerbate the extinction risk. Inbreeding depression remains a crucial area of research in conservation biology, ecology, and evolutionary biology. This study aims to elucidate the reproductive biology, inbreeding, and ID of *Paeonia decomposita* and to conserve, manage, and improve them better in the future.

**Results:**

*Paeonia decomposita* belongs to a xenogamous category and is partially self-compatible; moreover, it requires pollinators for seed production. Lately, the occurrence of pollination and pollinator limitations has affected the seed set. Low seed set primarily correlated with an abnormality of meiosis in the pollen mother cell, moderate to low genetic diversity, drought and extreme weather, pollinator limitation, or carpel space limit. One of the primary reasons for endangered mechanism in *P. decomposita* is the low seed set under natural conditions. The cumulative value of ID was positive, and outcrossed progeny outperformed selfed progeny.

**Conclusions:**

*Paeonia decomposita* requires pollinators to ensure seed production either through autogamy, geitonogamy, or allogamy. It is both allogamous and partially self-compatible, as well as a successful outcrosser. Inbreeding occurs frequently and results in ID, which imposes a potential threat to the survival of populations. Besides, it needs conservation via in situ and *natural return* methods.

## Background

*Paeonia* L. (Paeoniaceae) is a genus of 32 shrubbery and perennial herbs, mainly distributed in the northern hemisphere. The three sections in *Paeonia* include Section *Onaepia*, Section *Moutan*, and Section *Paeonia* (Hong 2010). Section *Moutan* comprises eight species, which are native and endemic to China (Hong 2010). Seed oil can be extracted from peony seeds, which contain fatty acid compositions, making them crucial woody oil crops at present (Zhang et al. 2015).

*Paeonia* seeds are big and mostly dispersed by gravity; thus, the transmission distance is very short. Besides, the pollen spread distance is very close (Luo et al. 1998; Hu et al. 2011). The distribution of individuals in natural habitat is gathered-type, and the number of individuals is low. The closer the distance, the higher the probability of successful pollination. However, the short distance of pollination and seed dispersion could result in close relative parents participating in mating. By the close offspring’s genetic distance, these individuals form a family in a population (Hu et al. 2011). Furthermore, long-term inbreeding would certainly result in homozygous genotype and the population will be prone to inbreeding depression (ID).

Typically, inbreeding (i.e., mating between relatives) decreases the fitness/vigor of progenies. To some extent, inbreeding could be detrimental to populations in a short time (Keller and Waller 2002; Charlesworth and Willis 2009). ID is explained through two famous hypotheses—partial dominance hypothesis and overdominance hypothesis (Charlesworth and Charlesworth 1987; Culley et al. 1999; Charlesworth and Willis 2009). To date, several empirical studies have shown that the majority of ID resulted from harmful recessive or partly recessive alleles, which are demonstrated in homozygosity of inbred individuals (Charlesworth and Charlesworth 1987; Crow 1993; Latter et al. 1995; Willis 1999; Charlesworth and Willis 2009). Some of the recessive alleles could be fatal in homozygosity, while a large part seems to be non-lethal alleles.

ID, the decline in survival and fertility of descendants in closely related individuals, was promptly recognized as the main hindrance to the evolution of autogamy (Darwin 1876; Charlesworth and Charlesworth 1987). ID is detrimental to plants, which has been evidenced by seed abortion, low germination rates, high seedling fatality, poor growth, and anthesis in descendants (Dudash and Fenster 2000; Oostermeijer et al. 2003; Vere et al. 2008). Furthermore, ID might occur at any phase of growth and development.

In small populations, as all partners are close relatives, they are, in particular, susceptible to inbreeding and ID (Keller and Waller 2002). As a consequence of increased inbreeding depression, small populations could be subjected to retarded growth rate, decreased population sizes, and even extinction (Hedrick and Kalinowski 2000; Keller and Waller 2002; Frankham 2005; O’Grady et al. 2006). Although biologists have been interested in ID since 1876 (Darwin, 1876), it remains active in conservation, pollination, and evolution.

*Paeonia decomposita* Handel-Mazzetti is one species of Section Moutan. In history, *P. decomposita* contained two subspecies—*P. decomposita* subsp *rotundiloba* and *P. decomposita* subsp *decomposita* (Hong 1997, 2010; Hong and Pan 1999; Hong et al. 2001); after extensive morphological studies and systematic analyses, both were raised as independent species (Hong 2011). As a shrub, *P. decomposita* can reach a height of 180 cm. Its seeds are black, glossy, broadly ellipsoid or globose, large and non-fleshy without specialized dissemination structure, making it not readily dispersed by birds and, thus, the spreading distance is very short.

*Paeonia decomposita* is endemic to China, distributed in the northwest of Sichuan Province, with sporadic and restricted distributions. The species has been recognized as a crucial woody oil crop, ornamental plant, and medicinal plant (its root-bark can be used as medicine, which is named “Danpi”). In addition, the population size is small and seedling recruitment is scant, making the species potentially apt to extinction. Based on the distribution, biological characteristics, and survival status, *P. decomposita* is an endangered (EN) plant in a threatened state (Hong et al. 2017).

Previously, some studies on reproductive biology have investigated some species of *Paeonia*, mainly concentrating on flora characteristics (Li et al. 2013; Yang et al. 2015), pollination (Grant 1964; Turpin and Schlising 1971; Schlising 1976; Luo et al. 1998; Zhou et al. 1999; Hong and Liu 2006; Li et al. 2014; Yang et al. 2015), breeding system (Li et al. 2013, 2014; Yang et al. 2015), pollinating insects behavior (Turpin and Schlising 1971; Schlising 1976; Luo et al. 1998; Li et al. 2014; Yang et al. 2015). Based on the related literature, this study adopts more comprehensive pollination treatments to measure self-compatibility and self-fertilization indices, percentage of pollinator limitation (PL) and pollination limitation (PPL), and ID. We performed one-sample *t* test to assess the effects of different pollination treatments, regression analysis, and correlation analysis. Currently, understanding of the reproductive biology, inbreeding, and ID of *P. decomposita* remains inadequate. Considering the great value in medicine, ornament, industry, and highly narrow distribution, this study aims to comprehensively investigate the reproductive biology, inbreeding, and ID to conserve, manage, and improve them better in the future.

## Methods

### Study site

The fieldwork was conducted from April to June 2017 in a fragmented forest near the suburb of Maerkang county of Sichuan Province, China (31° 52′ 46.945″–31° 52′ 54.368″ N, 102° 15′ 23.728″–102° 15′ 27.896″ E; 2700 m a.s.l.), where a large population of *P. decomposita* exists. In 2004, the population was much bigger than that now and the habitat was intact. With the time lapse, the natural habitats have been profoundly modified because of human activities such as fire, grazing, overly excavation, buildings, construction of hydroelectric power projects, and abusive specimen collection.

### Flowering phenology

Some plants of *P. decomposita* (*n* = 22) were arbitrarily marked before flowering to assess the flowering phenology. Then, we checked the flowering process of the population until opening the last flower of the last labeled plant. Then, the flowering individual’s percentage for each assessing day was calculated by dividing the number of marked plants with open flowers by the overall number of marked plants.

### Flowering duration, pollen viability, and stigma receptivity

In this study, 10 individuals were randomly selected and marked with a tag. Then, to calculate the flowering duration, two flowers on every tagged individual were marked before anthesis and observed until wilting daily. Then, floral buds from tagged individuals were bagged before flowering to avoid successive contamination of cross-pollen from the anthers to study the variation of pollen viability. All the flowers were packed in mesh bags, with 0.5 mm × 0.5 mm mesh, to prevent flower visiting without affecting the flower development. We harvested the pollens of 1–7 days after anthesis from different flowers. To evaluate the percentage of pollen viability, we used the I_2_-KI test (Li 2000) by counting > 500 pollen grains from nine individuals under a microscope (400×). Furthermore, stigma receptivity was determined using the H_2_O_2_ method (Dafni 1992).

### Pollen–ovule ratio

Mature buds were gathered just before anthesis and preserved in FAA to approximate overall pollen grains per flower. A specimen of the determined volume was located in a hemocytometer, in which the pollen grains were counted under a CX31 Olympus optical microscope (100×) and this magnitude was used to extrapolate the overall number of pollen grains per flower. The ovary was cut apart with a scalpel and the ovules were quantified by a stereomicroscope (120×). The pollen–ovule ratio (P/O) was evaluated by dividing the pollen grain number by ovule number per flower, as described by Cruden (1977).

### Pollination treatments

Pollination treatment was performed on randomly selected separate trees of *P. decomposita*. We used eight pollination treatment groups to determine the mating system and ID. Individual pollination treatment was performed on 40–80 flowers as follows: (1) natural pollination (control)—the flower buds were marked and left intact for open pollinating; (2) bagging without emasculation—the flower buds were marked and packed to investigate the autogamy (“autogamic pollination”); (3) bagging with emasculation—the flower buds were emasculated, marked, and packed to assess the apomixis; (4) bagging with mesh bag after emasculation—the flower buds were emasculated, marked, and packed with mesh bags to assess anemophily; (5) artificial geitonogamy—emasculated flowers were packed and pollinated with pollen of the flowers of the same shrub to measure the self-compatibility and potential ID; (6) artificial xenogamy—emasculated flowers were bagged and pollinated with pollen of the flowers of various plants > 10 m away; (7) emasculation without bagging—the flower buds were emasculated, labeled, and unbagged, exposed to pollinators, freely pollinated to detect efficiency of cross-pollination, outcrossing rates, and pollinator limitation levels; (8) hand supplemental pollination—unbagged flowers were handily pollinated with pollen of the flowers of dissimilar plants > 10 m away.

The emasculation manipulations were conducted before anthesis, and pollinations were performed when stigmas were receptive by brushing pollen over the stigmas until their surface was saturated. The bags were eliminated when withering the flowers to diminish the impact of bagging on fruit formation. When the follicle ripened and did not crack in autumn, fruits were harvested separately according to different treatments and placed in paper bags to allow fruits to break naturally. Next, the impact of each treatment was assessed according to the fruit set (ratio of fruits to treated flowers), seed set (ratio of seeds to the number of ovules), seed weight (g/1000 grain), percentage of carpels for seed set, number of seeds/fruit, and number of seeds/carpel.

### Related indices

We determined various indicators associated with the mating system using the findings achieved from hand-pollinating tests.

#### Self-compatibility and self-fertilization indices

Using those all apparently well-developed seeds, self-fertilization index (SFI) and self-compatibility index (SCI) were determined, as described by Lloyd and Schoen (1992). SCI is the seed set ratio between self-pollinated flowers and cross-pollinated flowers. SFI provides an approximation of a plant’s capacity to produce seeds with no pollen vector. SFI is the seed set ratio between spontaneous self-pollination flowers and cross-pollinated flowers.

#### Percentage of pollinator limitation (PL)

The degree of reproductive accomplishment restriction by an inadequate pollinator was evaluated using the following formula:

$$ PL = [(P_{X} - P_{E} ) \times 100]/P_{X}  $$

where *P*_*X*_ denotes the seed set of outcross-pollinated flower, and *P*_*E*_ denotes the seed set of the outcross-pollinated flower of emasculation without a bag (Castro et al. 2008).

#### Percentage of pollination limitation (PPL)

The degree of procreative accomplishment restriction by inadequate pollen delivery was defined as follows:

$$ PPL = [100 \times (PS - C)]/PS $$

where *PS* denotes for the seed set of pollen supplemental plants, and *C* represents the seed set of control plants (Jules and Rathcke 1999).

#### Inbreeding depression

The value of ID (*δ*) was calculated from four reproductive traits (number of seeds per fruit, fruit set, seed set, and weight of seed), as described by Charlesworth and Charlesworth’ method (1987), as follows:

$$ \delta = 1- W_{\text{s}} /W_{\text{o}} ,\quad {\text{when}}\;W_{\text{s}} \le W_{\text{o}} ;\quad {\text{or}}\;\delta = W_{\text{s}} /W_{\text{o}} - 1,\quad {\text{when}}\;W_{\text{s}} \ge W_{\text{o}} $$

where *W*_s_ denotes the average fitness of selfed progeny from hand self-pollination; *W*_o_ denotes the average fitness of manually outcrossed progeny from hand cross-pollination (Schemske and Lande 1985; Charlesworth and Charlesworth 1987).

The accumulative value of ID with the correlation among the data of the four parameters was calculated for the fitness ratio set of the four procreative features (fruit set, seed set, seeds per fruit, and seed weight). The seed germination was not included in measuring and evaluating the accumulative fitness in the present study, due mainly to the seed dormancy which was investigated pervasively in Section *Moutan* (Jing and Zheng 1999).

As described by Husband and Schemske (1996), we used the following formula:

$$ \delta = 1- [W{\text{s}}_{\text{f}} /W{\text{o}}_{\text{f}} \times W{\text{s}}_{\text{sf}} /W{\text{o}}_{\text{sf}} \times W{\text{s}}_{\text{s}} /W{\text{o}}_{\text{s}} \times W{\text{s}}_{\text{sw}} /W{\text{o}}_{\text{sw}} ] $$

where f is fruit set; sf is seeds per fruit; s is seed set; and sw is seed weight.

The values of ID range from − 1 to 1, where 0 signifies no ID, and positive values indicate the outcrossed offspring outperforming selfed offspring (ID), whereas the negative values simply the reverse style (Charlesworth and Charlesworth 1987).

### Statistical analyses

We calculated descriptive statistics for the P/O ratio, pollen viability, seed set, fruit set, and seed weight. All the analyses were performed using the program package SPSS 17 (SPSS Inc. Chicago, Illinois, USA) and Excel. In addition, we performed a one-sample *t*-test to assess the effects of different pollination treatments, using regression analysis and correlation analysis among seed weight, seed set (%), percentage of carpels for seed set, fruit set (%), number of seeds/fruit, number of seeds/carpel.

## Results

### Flowering phenology

Flowers opened sequentially from mid-April to late May each year; flowering duration of the population lasted for ≥ 43 days, and marginal differences were present between different years because of climate change. During the flowering period, a short blooming peak in late April existed, with > 50% of sampled plants in bloom.

### Floral lifespan, pollen viability, and stigma receptivity

A single flower was still open for some days up to felling off the stamens and corolla. The mean floral lifespan of a single flower was 7.75 days (*n* = 40 flowers). The anther dehiscence began occurring on the first day of the opening, and the average pollen viability was much higher (79.67% ± 1.61%; 1–7 after flowering). The viability reached a maximum (87.00% ± 1.12%) on the fourth day after blooming. While stigma was generally receptive on the second day after anthesis, it was rather weak and lasted for nearly 7 days.

### Pollen–ovule ratio

On average, *P. decomposita* flower produces 184,292,308 pollen grains and 73 ovules per flower. The P/O was 2,011,311 based on the category of Cruden (1977); (the ratio was calculated excluding the inviable pollen grains), the mating system of this species associated with the xenogamy class.

### Pollination treatment experiments

#### One-sample t-test

The *t* value of the one-sample *t*-test with SPSS in the seed weight, seed set (%), percentage of carpels for the seed set, number of seeds/fruit, number of seeds/carpel, and fruit set (%) were 4.259, 2.942, 3.575, 3.415, 3.991, and 4.087, respectively, and all the differences were significant (*P* < 0.05; Table 1).

**Table 1:** One-sample *t* test in seed weight (g/1000 grain), seed set (%), percentage of carpels for seed set, number of seeds/fruit, fruit set (%) and number of seeds/carpel in *P. decomposita*

Traits	T	df	Sig. (2-tailed)	Mean difference	95% confidence interval of the difference	
					Lower	Upper
Seed weight (g/1000 grain)	4.259	7	0.004	242.9088	108.0373	377.7802
Seed set (%)	2.942	7	0.022	13.0563	2.5622	23.5503
Percentage of carpels for seed set	3.575	7	0.009	49.3550	16.7131	81.9969
Number of seeds/fruit	3.416	7	0.011	7.6500	2.3540	12.9460
Fruit set (%)	4.087	7	0.005	59.6825	25.1497	94.2153
Number of seeds/carpel	3.991	7	0.005	2.6538	1.0815	4.2260

#### Effects of different pollination treatments

**Fig. 1:**
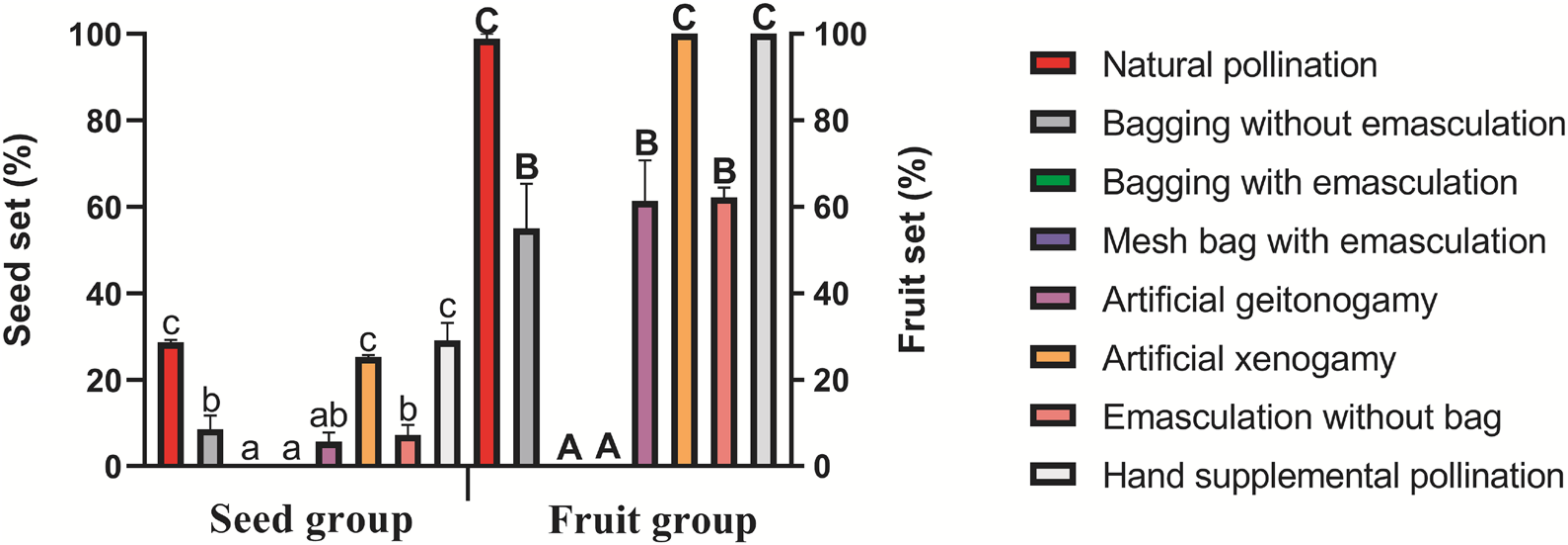
Seed set (%) and fruit set (%) of different pollination treatments in *P. decomposita*. Bars are means and vertical lines above bars are standard errors. The significant difference of seed set and fruit set were indicated in lowercase and bold capital, respectively

Table 2 presents detailed results of different pollination treatments. The pollen source markedly affected the seed set and fruit set, and no seed production was observed after bagging with emasculation and mesh bag with emasculation; this suggests that *P. decomposita* flowers were not able to apomixis and anemophily and, thus, relied on pollen transmission vectors to effective pollinating. The seed set and fruit set in bagging without emasculation (autogamy; 8.51% and 55%, respectively) and artificial geitonogamy (5.60% and 61.35%, respectively) were all low and similar, with no significant difference (*P* < 0.05), revealing a lack of a dichogamous system preventing the facilitated self-fertilization. In addition, artificial xenogamy and hand supplemental pollination treatment led to a higher fruit set (100% and 100%, respectively) and seed set (25.32% and 29.11%, respectively) compared with those in the bagging protection (Fig. 1). We noted no considerable difference in the average seed set among natural pollination, artificial xenogamy, and hand supplemental pollination; however, a markedly significant difference existed in the seed set between artificial geitonogamy, emasculation without bag, bagging without emasculation and natural pollination, artificial xenogamy, and hand supplemental pollination.

**Table 2:** Effects of different pollination treatments (mean ± SE) on several traits in *P. decomposita*

S. no.	Pollination treatments	Seed weight (g/1000 grain)	Seed set (%)	Percentage of carpels for seed set	Number of seeds/fruit	Fruit set (%)	Number of seeds/carpel
1	Natural pollination	290.03 ± 35.63^bc^	28.67 ± 0.59^c^	91.48 ± 3.57^c^	15.46 ± 0.88^c^	98.89 ± 1.11^c^	4.62 ± 0.23^c^
2	Bagging without emasculation	199.19 ± 80.74^b^	8.51 ± 3.22^b^	34.00 ± 12.61^b^	4.30 ± 1.71^b^	55.00 ± 10.41^b^	3.90 ± 2.11^bc^
3	Bagging with emasculation	0.00 ± 0.00^a^	0.00 ± 0.00^a^	0.00 ± 0.00^a^	0.00 ± 0.00^a^	0.00 ± 0.00^a^	0.00 ± 0.00^a^
4	Mesh bag with emasculation	0.00 ± 0.00^a^	0.00 ± 0.00^a^	0.00 ± 0.00^a^	0.00 ± 0.00^a^	0.00 ± 0.00^a^	0.00 ± 0.00^a^
5	Artificial geitonogamy	365.62 ± 15.38^c^	5.60 ± 2.2^ab^	41.33 ± 12.16^b^	5.73 ± 1.33^b^	61.35 ± 9.40^b^	1.94 ± 0.17^ab^
6	Artificial xenogamy	390.51 ± 23.16^c^	25.32 ± 0.43^c^	84.26 ± 4.05^c^	14.47 ± 1.34^c^	100 ± 0.00^c^	4.35 ± 0.30^bc^
7	Emasculation without bag	367.70 ± 60.56^c^	7.24 ± 2.27^b^	44.58 ± 7.67^b^	7.17 ± 1.64^b^	62.22 ± 2.22^b^	2.42 ± 0.35^abc^
8	Hand supplemental pollination	330.22 ± 6.56^c^	29.11 ± 4.11^c^	99.19 ± 0.81^c^	14.07 ± 1.66^c^	100 ± 0.00^c^	4.00 ± 0.54^bc^

Different lowercase letters in the same column indicate significant differences among pollination treatments (*P* < 0.05)

**Fig. 2:**
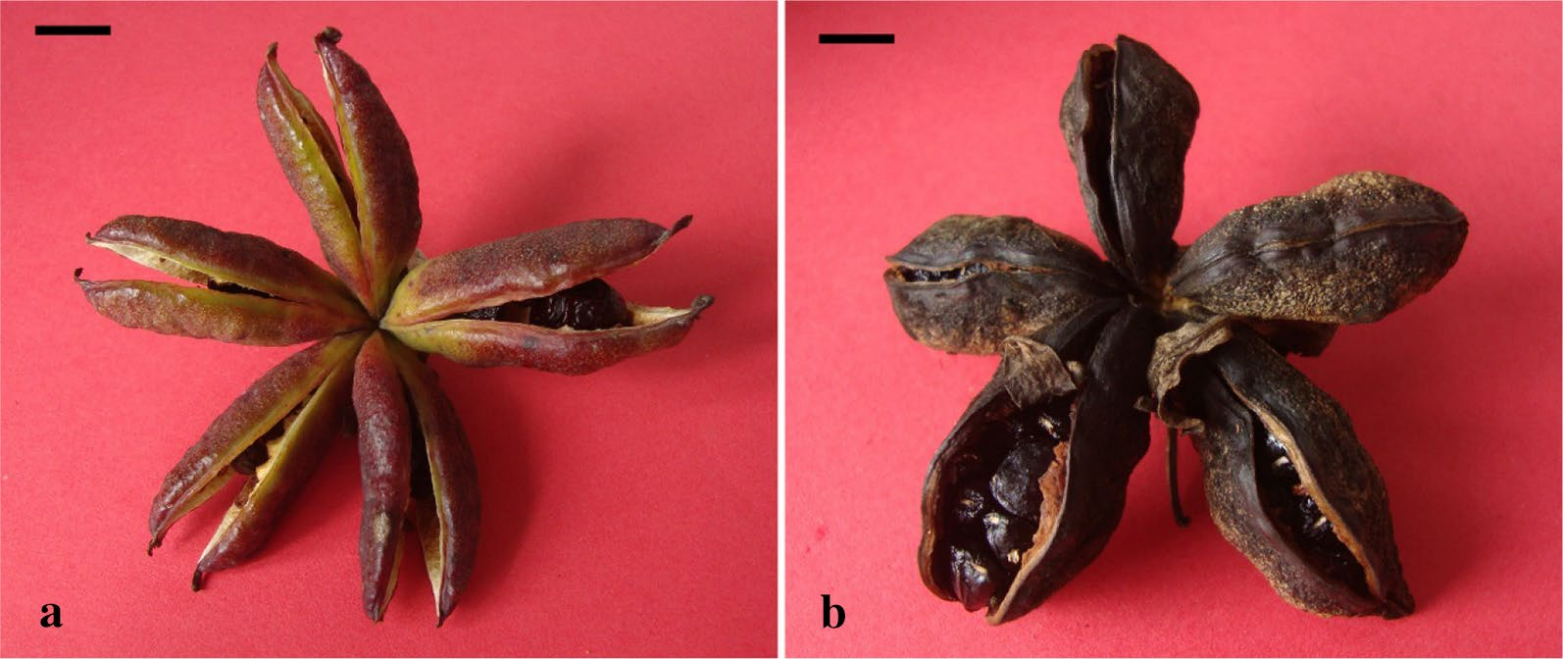
Fruit and seeds. **a** Artificial geitonogamy; **b** Artificial xenogamy. Scale bar = 1 cm

A majority of artificial geitonogamous flowers set were very few or without seeds, with an average of 5.73 seeds per fruit and 1.94 seeds per carpel (Table 2; Fig. 2a). Compared with the other treatments, the number of seeds per fruit and per carpel was relatively higher in artificial xenogamous (Fig. 2b), hand supplemental pollination flowers, as well as in natural pollination individuals (control plants). Moreover, we noted a significant difference in the number of seeds per fruit and percentage of carpels for seed set between artificial geitonogamy and artificial xenogamous, hand supplemental pollination flowers, and natural pollination individuals.

Regarding the seed weight, a significant difference was noted between bagging without emasculation and artificial geitonogamy, artificial xenogamy, emasculation without bag, and hand supplemental pollination (Table 2).

#### Correlation analysis

For six indices (seed weight, seed set, percentage of carpels for seed set, number of seeds per fruit, fruit set, and number of seeds per carpel), we observed a significant correlation (*P* < 0.05) between seed weight and percentage of carpels for seed set (*R* = 0.784), number of seeds/fruit (*R* = 0.769), and number of seeds per carpel (*R* = 0.743); a high correlation was observed between the seed weight and the fruit set (*R* = 0.880; *P* = 0.004). In addition, a really considerable relationship was noted at the 0.01 level between the seed set and the percentage of carpels for seed set, number of seeds/fruit, fruit set, number of seeds per carpel. Besides, no significant correlation was found between the seed set and the seed weight. Of note, we observed an extremely significant correlation at the level of 0.01 among other indices (Table 3).

**Table 3:** Correlation analysis among seed weight (g/1000 grain), seed set, percentage of carpels for seed set, number of seeds/fruit, fruit set, number of seeds/carpel in *P. decomposita*

index	Seed weight (g/1000 grain)	Seed set (%)	Percentage of carpels for seed set	Number of seeds/fruit	Fruit set (%)	Number of seeds/carpel
Seed weight (g/1000 grain)	–					
Seed set (%)	0.624	–				
Percentage of carpels for seed set	0.784^a^	0.971^b^	–			
Number of seeds/fruit	0.769^a^	0.972^b^	0.988^b^	–		
Fruit set (%)	0.880^b^	0.911^b^	0.975^b^	0.963^b^	–	
Number of seeds/carpel	0.743^a^	0.868^b^	0.893^b^	0.881^b^	0.936^b^	–

^a^Significant correlation at the 0.05 level

^b^Extremely significant correlation at the 0.01 level

#### Regression analysis

The regression analysis formula was obtained as follows:

$$ Y = - \;0.518 - 0.027X_{1} + 1.576X_{3} + 0.136X_{4} , $$

where *Y* denotes the seed set (%); *X*_1_ denotes the seed weight; X_3_ is the number of seeds/fruit; and X_4_ is the fruit set (%).

The main factors affecting the seed set are the number of seeds/fruit, seed weight, and fruit set (%). The order of influence on the seed set (*Y*) is the number of seeds/fruit, the weight of the seeds, and fruit set (%).

#### Self-compatibility and self-fertilization indices

The SCI value was 0.22, belonging to partial self-compatibility (SCI = 0.15–0.49; Ferrer et al. 2009). The SFI value was 0.34, over the threshold of 0.2 for self-compatibility (Lloyd and Schoen 1992).

#### PL

The fruit set (62.22%) and seed set (7.24%) of emasculation without bag were significantly lower than the artificial xenogamy treatment, suggesting the occurrence of pollinator limitation. Furthermore, a comparatively great pollinator limitation (71.41%) supported this issue.

#### PPL

Under natural circumstances, the seed set (28.67%) and fruit set (98.89%) were marginally lower than the hand supplemental pollination treatment, revealing the incidence of pollen limitation; however, the difference was not significant (*P* > 0.05). Of note, this finding was supported by the PPL value (1.51%).

#### ID

Based on the results of outcross pollination and self-pollination treatments in *P. decomposita*, the highest intensity of ID was obtained for the seed set (*δ* = 0.78), marginally higher for seeds/fruit (*δ* = 0.60), while relatively lower for fruit set (*δ* = 0.39) and seed weight (*δ* = 0.06) (Table 4). In this study, the cumulative value of ID was 0.9497. Furthermore, values > 0 reveal the further fitness of the outcrossed progeny compared to selfed progeny.

**Table 4:** Fitness of selfed and outcrossed progeny, and inbreeding depression coefficient (δ) of different traits

Plant traits	Outcrossed fitness	Selfed fitness	Inbreeding depression coefficient (δ)
Fruit set (%)	100	61.35	0.39
Number of seeds/fruit	14.47	5.73	0.60
Seed set (%)	25.32	5.60	0.78
Seed weight (g/1000 grain)	390.51	365.62	0.06

## Discussion

### Mating patterns

The flowering duration of the population was between April 12 and May 24 in 2017. The flowering period of a single flower was usually between 6 and 9 days (average: 7.75 days). In plants, flowers are the only parts that directly attract the pollinator; thus, its functional and morphological features could affect the plant’s procreative accomplishment. During the early flowering period, only a few other plants were in bloom, thereby guaranteeing pollinators’ pollination on *P. decomposita*. The P/O ratio suggests that *P. decomposita* is xenogamy, similar to *P. delavayi* (Li et al. 2013, 2014) and *P. californica* (Schlising 1976). The results of hand-pollination experiments, self-fertilization, and self-compatibility indices demonstrated that *P. decomposita* required pollinators’ visitation to form seed, as there was no seed after pollinator exclusion (Table 2), which belonged to the xenogamous category, partial self-compatibility. These findings corroborated the type of predicted P/O value. *P. decomposita* is not capable of apomixes and anemophily, and flowers advanced rewards (pollen) to attract the pollinators to transport their male gametes (Dafni et al. 2005).

### Seed set

Pollen quality and availability were the two main factors of female reproductive accomplishment (Griffin and Barrett 2002). Pollination quality is determined by pollinators’ behavior, which can be affected by plant density (Grindeland et al. 2005). Reportedly, pollinator limitation and pollen limitation are extensive, especially in species pollinated by animals (Ashman et al. 2004). Co-flowering plants could result in the competition of pollinators, incremented heterospecific pollen provision, and/or stigma clogging by heterospecific pollen (Gross 1996). For *P. decomposita*’s population, other species also starts flowering during its peak flowering duration, competing for latent pollinators, and decreasing the visitation rate to *P. decomposita*. In the treatment of emasculation without bag, the visit number and visit rate of insects markedly decreased because of removed and lost attraction to insects by stamen and petal. Thus, pollinator limitation occurred and resulted in a significantly lower seed set (7.24%). In addition, hand supplemental pollination did not considerably increase seed production (0.44%) compared with natural pollination. As the individual distribution of *P. decomposita* is sporadic and narrow in small and fragmented range, pollen resources for pollination could be restricted. In addition, the resource allocation and available resources play a vital role in the ultimate female reproductive success (Wesselingh 2007). Thus, the occurrence of pollination limitation and pollinator limitation during the pollination process affected the fruit set and seed set to some extent in this species.

The results of pollination revealed that *P. decomposita* could produce seed by self-pollination; however, the seed set was very low [only 5.60% in artificial geitonogamy, lower than that in Yang et al. (2015) study, 11.04%; 8.51% in autogamy, higher than that in Yang et al. (2015) study, 4.06%], which was significantly lower than artificial xenogamy (25.32%), and the difference was significant (*P* < 0.05), suggesting the existence of an auto-incompatibility structure. The incidence of auto-incompatibility structure would enhance outcrossing and avoid ID (Silva and Goring 2001). While the seed set in artificial xenogamy [25.32%, higher than that in Yang et al. (2015) study, 18.33%] was lower than that in natural pollination [28.67%, higher than that in Yang et al. (2015) study, 23.76%], and no considerable difference existed in their seed set (*P* > 0.05). Usually, seed set of natural pollination was very low (< 30%) in the studied species (Schlising 1976; Luo et al. 1998; Hong and Liu 2006; Yang et al. 2015; this study) of *Paeonia* genus, except for *P. delavayi* (77.8%; Li et al. 2013, 2014).

While 4.62 mature seeds existed per carpel of natural pollination, 11.6 ovules per carpel could not fertilize and form seeds or abort in *P. decomposita*. However, there were 4.2 seeds per carpel in *P. californica* (Schlising 1976) and 2.1 seeds per carpel in *P. jishanensis* (Zhou et al. 1999). The low seed set in this species largely correlated with some factors, including the abnormality of meiosis in pollen mother cells (Wang and Zhang 2008), moderate genetic diversity (unpublished data), drought and extreme weather (continuous rain in 2017, drought in 2018), pollinator limitation, carpel space limit (Stebbins and Ellerton 1939; Walter 1942; Schlising 1976). The low seed set in natural condition was one of the main reasons for the endangered mechanism in *P. decomposita*.

### Inbreeding depression

Inbreeding is not avoidable in small isolated populations (Frankham et al. 2003), and it is supposed to exacerbate the risk of extinction in endangered groups (Brook et al. 2002). Reportedly, levels of outcrossing are lower for plant populations in fragmented or disturbed environments compared with the undisturbed habitats (Ward et al. 2005; Coates et al. 2007). As inbreeding could be resulting from subdividing the population (Keller and Waller 2002), the population subdivision level is a factor leading to the difference in the inbreeding level. In this study, the impact of isolation and population size could not be assessed owing to the population delimitation subjectivity. Notably, the pollinators’ foraging behavior is affected by the density of plant population (Field et al. 2005). Thus, the plants’ spatial distribution in a population, perhaps, affects the outbreeding level via the pollinators’ visitation arrangement.

To the best of our knowledge, this is the first study to investigate the inbreeding levels and extent of ID on an endangered species in the greatly fragmented forestry ecosystem in *Paeonia*. *P*. *decomposita* is prone to ID because reproduction happens between close relatives or by individual owns. The seed number was considerably lower in the self-pollination, suggesting that a high amount of unfit inbred progeny harboring deleterious alleles are already eliminated over-fertilizing or at the early seed development phase.

Based on the outcomes of self-pollination and outcross-pollination treatments, the ID value of four traits (seeds per fruit, fruit set, seed weight, and seed set) of *P. decomposita* was different. The accumulative value of ID was high and positive (0.9497) and outcrossed progeny outperformed selfed progeny.

### Conservation and management implications

Lately, human activities have markedly influenced natural habitats of *P*. *decomposita*. Owing to important ornamental, medicinal, and oil value, local villagers excavate its roots and collect its seeds for personal benefits. Moreover, the massive excavation and collection make it more difficult to regenerate.

Inbreeding frequently occurs in *P*. *decomposita* and results in a considerable ID levels. In addition, inbreeding and ID are a latent risk to the survival of populations and, thus, should be considered in planning the conservation actions. It would be especially significant to investigate the necessity of genetic management, inbreeding levels, and the occurrence rate of ID in markedly endangered species in small forest fragments.

This study offers practical, useful, and valuable information to guide recovery efforts for *P. decomposita* populations. Executing an active program to restore this species is warranted, given the extensive habitat destruction of human activities in the past and the restricted size of the few remaining *P. decomposita* populations. Besides, restoration efforts are most probably to succeed in areas with a large population of plants, especially if populations contain plants gathered from a few existing sites.

Hence, conservation approaches for *P. decomposita* should focus on the following features: (1) local government should establish some small nature reserves to limit the exploitation and govern the damage to natural habitats and enhance the original habitats’ recovery; (2) large-scale cultivation should be carried out to cater to market demand and minimize damages to wild species; and (3) breeding technology should be improved and more saplings should be cultivated. In addition, they should be introduced back into the original habitats to increase the population density and offer more pollen sources. Perhaps, this study would provide a basis for better conservation, management, and improvement in the future.

## Conclusions

Like most other *Paeonia* species, *P. decomposita* requires pollinators to ensure seed formation both via autogamy (pollinating a flower by its own pollen), geitonogamy (pollinating within flowers of the same plants), or allogamy (pollinating within flowers from various individuals), and appeared to be both allogamous and partial self-compatibility and, thus, a successful outcrosser. *P. decomposita* is not capable of apomixes and anemophily, and flowers attract pollinators by pollen. In addition, the number of seeds per carpel and seed set in this species is all low. Besides, inbreeding occurs frequently in *P*. *decomposita* and results in ID, creating a latent threat to the population survival requiring conservation via in situ and *natural return* methods.

## Data Availability

The datasets used and/or analyzed during the current study are available from the corresponding author on reasonable request.

## Abbreviations

ID: inbreeding depression
P/O: pollen–ovule ratio
PL: percentage of pollinator limitation
PPL: percentage of pollination limitation
SCI: self-compatibility index
SFI: self-fertilization index
